# Asian business and management: review and future directions

**DOI:** 10.1057/s41291-022-00209-y

**Published:** 2022-11-04

**Authors:** Fabian Jintae Froese, Ashish Malik, Satish Kumar, Saumyaranjan Sahoo

**Affiliations:** 1 University of Goettingen, Platz der Goettinger Sieben 5, 37073 Goettingen, Germany; 2 Newcastle Business School, Callaghan, NSW Australia; 3 Malaviya National Institute of Technology, Jaipur, India; 4 Jaipuria Institute of Management, Jaipur, India

**Keywords:** Bibliometrics, Review, Bibliometric analysis, Asian business and management

## Abstract

This century has been proclaimed the Asian century, as industrialised countries such as Japan, Singapore, and South Korea, along with rapidly emerging nations such China and India, have contributed to worldwide economic growth. In response, research has analysed the reasons why Asian business and management have found such success. Based on a bibliometric analysis of *Asian Business & Management* (ABM), a premier journal devoted to Asian management, here we examine the performance of the research constituents, social structure, and intellectual structure of 331 scholarly papers, which sheds light on the growing influence of ABM through six major knowledge clusters: corporate social responsibility; business management in emerging markets; corporate governance; internationalization; political and business ties; and organization culture and performance. Temporal analysis reveals the emergence of strategy and human resource management as a distinct knowledge cluster and the increasing importance of China as a research context and producer. Based on this analysis, we propose future research directions.

## Introduction

The centers of economic and political powers have shifted throughout history. While India and China were the two dominant economies until the year 1820, the industrial revolution shifted the balance of power to Europe and North America (Mahbubani, 2011). However, more recently, we witnessed a rebalancing of economic activity back to Asia, triggering a rethiniking of strategic goals of the West for Asia (Mahbubani, 2008, 2011). Highly industrialized Asian countries, for example, such as Japan, Singapore, and South Korea, along with rapidly re-emerging nations such China and India, have contributed to worldwide economic growth. China alone has lifted more than 800 million people out of poverty over the last few decades (Worldbank, 2022). Toyota, Samsung, Alibaba, and Infosys have become world-leading companies, setting new trends in their respective industries. As such, this century has been proclaimed as the Asian century.

In response, a growing body of scholarly literature has analyzed the reasons why Asian business and management have become so successful. In line with this development, Harukiyo Hasegawa founded the journal *Asian Business & Management* (ABM) in 2002, which has been recognized as a leading journal in the fields of business and management with a focus on the Asian region. ABM is celebrating its twentieth anniversary in 2022; to commemorate the event, we conducted a bibliometric analysis of the journal, which also serves as an excellent reflection of the research landscape in the area of business and management in Asia. Bibliometric methodology provides an unbiased review of the journal through quantitative assessment in a variety of areas (Donthu et al., 2022; Gammelgaard et al., 2020; Kataria et al., 2021), including content and pattern relating to publications, citations, contributors (i.e., authors, institutions, countries, and publishing outlets), geographical focuses, and scholarly contributions (themes and topics). Given ABM’s accomplishments and reputation over the preceding two decades, the current investigation attempts to answer the following research questions (RQs):

### RQ1

What are the publication and citation trends of ABM?

### RQ2

Which authors, institutions, and countries have contributed the most to ABM?

### RQ3

Which are the most-cited articles and journals for ABM?

### RQ4

What are the collaboration patterns of ABM’s contributors?

### RQ5

What are the most prevalent themes and topics in ABM? How have they evolved over time?

### RQ6

What are the emerging research avenues that ABM and its prospective contributors may pursue in the future?

The remainder of this article is structured as follows. The methodology used in this study is discussed in the next section, followed by the results of the performance analysis and science mapping in the following sections. Bibliometric analysis and the selected articles in the 20-year anniversary issue of ABM serve as the basis for inspiration for future research directions, which we discuss at the end of this article.

## Methodology

### Data collection method and procedure

Before describing the methodology of the bibliometric analysis, we briefly introduce relevant metrics in science and apply them to ABM. The popularity of the journal among academia may be gauged using established journal-related performance indicators such as CiteScore (CS) and impact factor (IF). Scopus reports the CS of journals based on the last 4 years of bibliographic records from the Scopus database, while Clarivate Analytics reports the IF of journals based on the latest 2 years of bibliographic records from the Web of Science (WoS) database (Donthu et al., 2022; Waris et al., 2021). ABM was included in Clarivate Analytics (its predecessor) in 2007 and Scopus in 2008. Scopus assigns ABM a CS of 5.500 for 2021, which means that articles in the journal earned an average of 5.500 citations between 2017 and 2020 from other journals indexed in Scopus database, while Clarivate Analytics gives ABM an IF of 4.130, which indicates that articles in the journal earned an average of 4.135 citations per published article between 2019 and 2020 from other journals indexed in the WoS database. ABM’s source normalized impact per paper (SNIP) in 2021 is 1.371, implying that the journal’s average citations are 1.371 times greater than the average citations of other journals in its subject domain, which are indexed in Scopus. ABM has also been recognized as a quality journal in the business management domain, with a rating of “2” in the *2021 Academic Journal Guide* endorsed by the Chartered Association of Business Schools (CABS).

We combed the Scopus database, one of the largest collections of peer-reviewed scientific literature (Gammelgaard et al., 2020; Kataria et al., 2021; Pattnaik et al., 2021; Sureka et al., 2020), seeking information on articles published in ABM in the two decades since the journal’s inception, to generate the bibliographic metadata for this study. On September 8, 2022, the keyword phrase “Asian Business and Management” was entered as a search query in the Scopus Database, with the “source title” option selected, yielding a search result of 331 articles after we excluded duplicates and editorials, with 2008 being the initial Scopus-indexed published year. A similar query conducted into the WoS database yielded similar article statistics with an additional 24 articles in 2007. Due to the comprehensive nature of bibliographic data, we opted to leverage the Scopus Database for final data extraction, which provided a result of 331 articles.

**Fig. 1:**
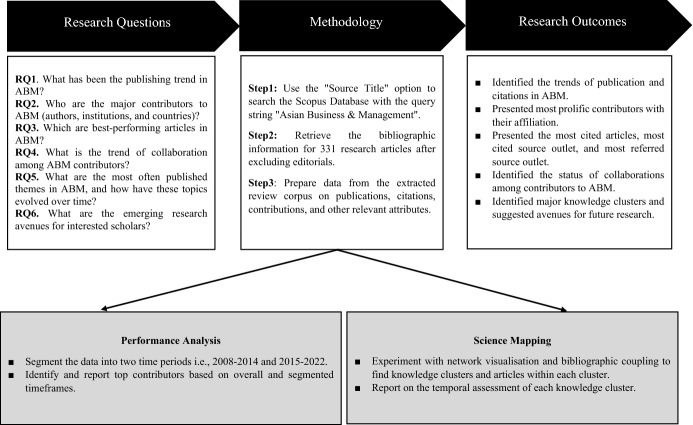
Research design

Bibliometrics is a field of information and library sciences that use statistical methods to study the bibliographic data of an extracted review corpus (Donthu et al., 2022; Gammelgaard et al., 2020; Kataria et al., 2021); further, the evaluation outcome using accessible bibliometric software is either a tabular synthesis or graphical representation of a review corpus to demonstrate the evolution of a research field of interest. The use of bibliometric analysis in the academic areas of business management and social science is becoming increasingly common (Donthu et al., 2022; Kumar et al., 2022; Sahoo, 2021), with most published work of this kind containing study of coverage patterns, comprehensive introspection, and unbiased observations (Dana et al., 2021; Varma et al., 2022; Waris et al., 2021). This fundamental assumption considers citation data to be reliable indicators of the advancement of the study area, illuminating the roles played by influential authors and their institutions and mapping the underlying structures in scholarly outputs and their interconnections (Prakash et al., 2022; Sureka et al., 2020; Viglia et al., 2022). In keeping with previous research (Donthu et al., 2022; Varma et al., 2022; Viglia et al., 2022), this study employs two unique types of bibliometric analysis: performance and scientific mapping (Donthu et al., 2021). We divided the retrieved bibliographic data into two seven-year (approx.) intervals, 2008–2014 and 2017–mid-2022, to provide a clear picture of ABM’s publication history. This method was chosen in accordance with previous research in the field. Figure 1 highlights the current investigation’s research design and assessment methodology.

### Data analysis method and procedure

This research examines the performance of contributors in terms of (1) publishing and citation trends; (2) leading authors, institutions, and countries; (3) most-cited articles; (4) leading journals referenced by ABM’s articles; and (5) leading journals citing ABM’s articles, shedding light on ABM’s influence and productivity (Donthu et al., 2021; Varma et al., 2022; Viglia et al., 2022). To assess ABM’s performance, several metrics were used (“Appendix”), including total publications (TP), total cited publications (TCP), total citations (TC), citations per publications (TC/TP), citations per cited publications (TC/TCP), citations per active year since publication (C/Y), *h-index*, and IF and CS. The quantitative existence of research constituents such as authors, institutions, and countries is defined by TP, whereas the quantifiable scholarly influence of these elements is designated by TC (Donthu et al., 2021, 2022; Gammelgaard et al., 2020; Pattnaik et al., 2021). TCP counts influential or referenced articles, providing a logical justification for using TC/TCP rather than TC/TP (Donthu et al., 2022; Kataria et al., 2021; Varma et al., 2022). The *h-index*, like IF and CS, is a similar impact-measuring metric defined as the maximum value of *h* such that the given author/journal has written at least *h* articles, each of which has been cited at least *h* times (Gammelgaard et al., 2020; Waris et al., 2021).

Network visualization was utilized in scientific mapping to uncover the main knowledge clusters that constitute ABM’s social and intellectual structure. Drawing on previous research (Donthu et al., 2022; Kataria et al., 2021; Varma et al., 2022), the collaboration pattern among research constituents was found using co-authorship analysis, while the knowledge cluster was established using the co-occurrence of the author keyword method. It is fairly obvious that authors select keywords to represent the topics and themes that lie at the heart of their academic work and also to increase their exposure among interested researchers (Sureka et al., 2020; Viglia et al., 2022). As a consequence, utilising keyword co-occurrence analysis to explore makes sense (Emich et al., 2020; Pattnaik et al., 2021). Synonymous keywords are also rendered homogenous in the retrieved data set. Articles within each thematic cluster were identified using the bibliographic coupling approach, which uses references to establish similarity across publications, supplementing the identification of thematic cluster via the co-occurrence of author keyword analysis (Aria & Cuccurullo, 2017; Dana et al., 2021; Sureka et al., 2020). The degree of similarity, as shown by the robustness of the citation relationships between them, increases as the number of shared references increases (Donthu et al., 2021). Next, a content analysis method was chosen to articulate the description of each thematic cluster and to recommend future research possibilities. We conducted the review evaluation of ABM using Microsoft Excel, Bibliometrix-R, and Gephi software.

## Performance analysis of Asian business & management

### Data year-wise patterns of publications

As a result of the activities mentioned in the methodology section, the extracted review corpus comprises 331 articles published in ABM between 2008 and 2022, a synopsis of which is shown in Table 1 (RQ1), which also shows that the number of publications (TP) in ABM has been around 20 between 2008 and 2013, around 15 between 2014 and 2019, and increased sharply from 2020, with the highest number of publications being 48 in 2022. Likewise, total citations, cumulative citations, and cites per publications increased rapidly in recent years.

**Table 1:** Annual trend of publications and citations in ABM between 2008 and 2022

Year	*TP*	*CTP*	*TC*	*CTC*	*CTC/CTP*
2008	21	21	2	2	0.095
2009	20	41	11	13	0.317
2010	17	58	28	41	0.707
2011	17	75	60	101	1.347
2012	24	99	59	160	1.616
2013	25	124	107	267	2.153
2014	14	138	99	366	2.652
2015	17	155	126	492	3.174
2016	16	171	146	638	3.731
2017	15	186	163	801	4.306
2018	15	201	201	1002	4.985
2019	16	217	229	1231	5.673
2020	29	246	365	1596	6.488
2021	48	294	510	2106	7.163
2022	37	331	537	2643	7.985

*TP* total publications, *CTP* cumulative total publications, *TC* total citations, *CTC* cumulative total citations, *CTC/CTP* cites per publication

Table 2 provides a ranking of the most prolific authors in ABM between 2008 and 2022. The ranking provides an overview of absolute and relative publications as well as citations but does not distinguish between single or co-authored papers. Tomoki Sekiguchi from Kyoto University leads the ranking in terms of total publications, as he published seven articles in ABM. He is followed by Martin Hemmert from Korea University with six publications and Jie Wu from Aberdeen University with five publications. Yipeng Liu from the University of Reading leads the ranking in terms of total citations.

**Table 2:** Authors with the highest contribution to ABM between 2008 and 2022

Authors, Current Affiliation & Country	*TP*	*TCP*	*TC*	*TC/TP*	*TC/TCP*	*h*
T. Sekiguchi, Kyoto University, Japan	7	5	70	10.00	14.00	4
M. Hemmert, Korea University, South Korea	6	6	50	8.33	8.33	4
J. Wu, University of Aberdeen, United Kingdom	5	5	51	10.20	10.20	4
F.J. Froese, University of Göttingen, Germany	4	4	51	12.75	12.75	3
C. Lakshman, Florida Atlantic University, United States	4	3	20	5.00	6.67	3
D. Shin, Yonsei University, South Korea	4	4	15	3.75	3.75	3
J. Cho, Kyungpook National University, South Korea	3	3	19	6.33	6.33	1
A.M. Colpan, Kyoto University, Japan	3	2	7	2.33	3.50	2
J.J. Cordeiro, State University of New York, United States	3	3	26	8.67	8.67	3
A. Giroud, University of Manchester, United Kingdom	3	3	39	13.00	13.00	2
I.M. Jawahar, Illinois State University, United States	3	2	18	6.00	9.00	1
I. Jeong, Korea University, South Korea	3	2	8	2.67	4.00	1
E. Kim, Pusan National University, South Korea	3	2	21	7.00	10.50	2
J. Lee, Kyungpook National University, South Korea	3	3	19	6.33	6.33	1
Y. Liu, University of Reading, United Kingdom	3	3	141	47.00	47.00	3
M.H. Ogasavara, Escola Superior de Propaganda e Marketing, Brazil	3	2	24	8.00	12.00	2
P. Pananond, Thammasat University, Thailand	3	3	38	12.67	12.67	3
J.H. Rhee, Korea University, South Korea	3	3	42	14.00	14.00	3
F. Robins, University of Adelaide, Australia	3	3	29	9.67	9.67	2
H. Sakawa, Nagoya City University, Japan	3	3	26	8.67	8.67	3
D. Sutherland, Durham University, United Kingdom	3	3	50	16.67	16.67	3
N. Watanabel, Nagoya City University, Japan	3	3	26	8.67	8.67	3
T. Yoo, Hankuk University of Foreign Studies, South Korea	3	3	35	11.67	11.67	3

Authors with minimum number of 3 articles, *TP* total publications, *TCP* total cited publications, *TC* total citations, *TC/TP* cites per publication, *TC/TCP* cites per cited publication, *h* h-index

Korea University (South Korea) has the highest contribution to ABM with 21 publications and *h-index* score of 9. The University of Reading (United Kingdom) has the highest TC/TP score of 27. The top researchers, as mentioned above, greatly contributed to the success of those institutions (Table 3).

**Table 3:** Institutions with the highest contribution to ABM between 2008 and 2022

Institutions	*TP*	*TCP*	*TC*	*TC/TP*	*TC/TCP*	*h*
Korea University, South Korea	21	20	189	9.00	9.45	9
Zhejiang University, China	10	9	91	9.10	10.11	6
Yonsei University, South Korea	10	9	58	5.80	6.44	5
Gothenburg University, Sweden	9	8	82	9.11	10.25	6
Tongji University, China	9	8	126	14.00	15.75	7
National Taiwan University, Taiwan	8	8	35	4.37	4.37	5
Tsinghua University, China	7	6	50	7.14	8.33	3
Kyoto University, Japan	7	6	18	2.57	3.00	5
University of Reading, United Kingdom	6	6	162	27.00	27.00	5
Sungkyunkwan University, South Korea	5	5	68	13.60	13.60	4
Seoul National University, South Korea	5	5	105	21.00	21.00	4
Xi'an Jiaotong University, China	5	5	37	7.40	7.40	5
Macquarie University, Australia	5	5	89	17.80	17.80	3
University of Macau, China	5	5	51	10.20	10.20	4
Osaka University, Japan	5	5	89	17.80	17.80	4
Keio University, Japan	5	5	89	17.80	17.80	3
University of Goettingen, Germany	5	5	86	17.20	17.20	4
Stockholm University, Sweden	5	5	52	10.40	10.40	4

Institutions with minimum number of 5 articles, *TP* total publications, *TCP* total cited publications, *TC* total citations, *TC/TP* cites per publication, *TC/TCP* cites per cited publication, *h* h-index

Table 4 displays the key indicators of performance for the leading countries that contributed to ABM from 2008 and 2022 (RQ2). China appears as the most prolific and influential country contributing to ABM across various parameters, including total publications (TP = 96), total cited publications (TCP = 78), total citations (TC = 797), and *h-index* (*h* = 17). Following China are the United States (TP = 72, TCP = 64, TC = 764, *h* = 16) and South Korea (TP = 59, TCP = 53, TC = 467, *h* = 14). The Netherlands tops the list on the TC/TP criteria with a score of 19.16, while Singapore leads on the TC/TCP criterion with a score of 20.88. This suggests that authors from the Netherlands and Singapore were able to produce more high-impact articles.

**Table 4:** Countries with the highest contribution to ABM between 2008 and 2022

Countries	*TP*	*TCP*	*TC*	*TC/TP*	*TC/TCP*	*h*
China	96	78	797	8.30	10.22	17
United States	72	64	764	10.61	11.94	16
South Korea	59	53	467	7.92	8.81	14
Japan	42	39	377	8.98	9.67	12
United Kingdom	34	34	456	13.41	13.41	13
Australia	20	20	253	12.65	12.65	10
Taiwan	20	19	134	6.70	12.65	7
Germany	16	15	211	13.19	14.06	10
Sweden	15	14	145	9.67	10.36	8
India	12	11	67	5.58	6.09	5
Canada	11	11	91	8.27	8.27	8
Singapore	10	8	167	16.70	20.88	5
France	9	9	78	8.67	8.67	6
Hong Kong	6	4	27	4.50	6.75	2
Macao	6	5	51	8.50	10.20	4
Malaysia	6	6	58	9.67	9.67	4
Netherlands	6	6	115	19.16	19.16	6
Pakistan	6	5	110	18.33	22.00	5
Thailand	6	6	72	12.00	12.00	5

Countries with minimum number of 6 articles, *TP* total publications, *TCP* total cited publications, *TC* total citations, *TC/TP* cites per publication, *TC/TCP* cites per cited publication, *h* h-index

Table 5 depicts the temporal distribution of the top authors, institutions, and countries spanning two time points: 2008–2014 and 2015–2022. According to the temporal distribution, no authors produced more than three articles in the first time phrase (2008–2014), while five authors contributed five or more articles in the second time phrase (2015–2022). On the influential metrics (TC), Sarkis and Zhu with 79 citations from two articles are the most impactful authors in the first time frame (2008–2014), while Liu with 152 citations from four articles is the most impactful author in the second time frame (2015–2022). While National Taiwan University contributed the most articles in the first time frame (seven articles), Korea University contributed the most articles in the second time frame (17 articles). The United States (TP = 27, TC = 402) and Japan (TP = 21, TC = 237) come in top and second, respectively, on the criteria of TP and TC when comparing the contribution and performance of countries to and in ABM from 2008 to 2014. In the second time period (2015 to 2022), China emerges as the number-one contributor to ABM with a TP value of 83 articles and as an influential contributor to ABM with a TC value of 605 citations.

**Table 5:** Temporal distribution of contributing authors, institutions, and countries to ABM

2008–2014 (138 Documents)			2015–2022 (193 Documents)		
*Author*	*TP*	*TC*	*Author*	*TP*	*TC*
T. Yoo	3	36	J. Wu	8	66
F. Robins	3	29	T. Sekiguchi	6	64
J. Sarkis	2	79	Y. Yang	6	28
Q. Zhu	2	79	J. Cho	5	29
F. Jiang	2	49	M. Hemmert	5	42
K. Lee	2	40	Y. Liu	4	152
J.H. Rhee	2	33	D. Ryu	4	94
Y. Hoshino	2	30	Y. Wang	4	10
J. Jaussaud	2	30	X. Chen	3	20
M. Rhee	2	27	J.J. Cordeiro	3	26
S. Söderman	2	26	X. He	3	44
Y. Yang	2	26	H. Sakawa	3	26
H. Dolles	2	24	D. Sutherland	3	50
M.J. Teng	2	16	N. Watanabel	3	26
C. Lakshman	2	14	L. Zhang	3	14
H. Chen	2	11	F.J. Froese	3	34
A.M. Colpan	2	7	E. Kim	3	21
T. Hikino	2	7	J. Lee	3	21
B. Andreosso-O'Callaghan	2	3	J. Li	3	12
J. Park	2	1	D. Shin	3	12
M.A. Witt	2	1	X. Wang	3	10
			I.M. Jawahar	3	18
			I. Jeong	3	8
			Y. Zhou	3	3

2008–2014 (138 Documents)			2015–2022 (193 Documents)		
*Institution*	*TP*	*TC*	*Institution*	*TP*	*TC*
National Taiwan University	7	60	Korea University	17	142
Gothenburg University	5	67	Zhejiang University	10	91
Stockholm University	5	52	Tongji University	8	117
Korea University	4	47	Xi’an Jiaotong University	7	61
Macquarie University	4	86	Yonsei University	7	30
Hankuk University of Foreign Studies	3	35	Kyoto University	6	21
Seoul National University	3	77	Tsinghua University	6	48
University of Hawaii at Manoa	3	35	Jilin University	5	21
Yonsei University	3	29	University of Reading	5	149
National University of Singapore	3	49	University of Macau	5	51
University of South Australia	3	42			

2008–2014 (138 Documents)			2015–2022 (193 Documents)		
*Country/Region*	*TP*	*TC*	*Country/Region*	*TP*	*TC*
United States	27	402	China	83	605
Japan	21	237	United States	45	371
South Korea	18	213	South Korea	41	261
Australia	14	231	United Kingdom	24	307
China	13	196	Japan	21	155
Taiwan	13	96	Germany	10	125
Sweden	10	119	Canada	7	42
United Kingdom	10	151	India	7	35
France	8	76	Taiwan	7	38
Germany	6	101	Australia	6	23
			Macao	6	51

List contains leading 10 contributors in each category, *TP* total publications, and *TC* total citations

Table 6 presents the top 15 most-cited articles out of 331 articles published in ABM (RQ3). The article “The challenges and opportunities of a global health crisis: the management and business implications of COVID-19 from an Asian perspective” by Liu et al. (2020) has garnered 101 citations and has the highest TC/Y score of 50.50 on the list. It is noteworthy that many highly cited articles were published in the last 3 years.

**Table 6:** Most-Cited Articles Published in ABM between 2008 and 2022

TC	Author	Title	Year	TC/Y
101	Y. Liu, J.M. Lee, and C. Lee	*The challenges and opportunities of a global health crisis: the management and business implications of COVID-19 from an Asian perspective*	2020	50.50
57	P. Shapira and J. Wang	*From lab to market? Strategies and issues in the commercialization of nanotechnology in China*	2009	4.38
52	N.A. Khan, A.N. Khan, and S. Gul	*Relationship between perception of organizational politics and organizational citizenship behavior: testing a moderated mediation model*	2019	17.33
52	Q. Zhu, J. Sarkis, and Y. Geng	*Barriers to environmental* *ly friendly clothing production among Chinese apparel companies*	2011	4.73
42	J. Mathews	*China, India and Brazil: Tiger technologies, dragon multinationals and the building of national systems of economic learning*	2009	3.23
41	T. Sekiguchi, F. Jintae Froese, and C. Iguchi	*International human resource management of Japanese multinational corporations: Challenges and future directions*	2016	6.83
39	J. Wu and S. Si	*Poverty reduction through entrepreneurship: incentives, social networks, and sustainability*	2018	9.75
38	L. Wong	*Corporate social responsibility in China: Between the market and the search for a sustainable growth development*	2009	2.92
37	D.-S. Cho, H.-C. Moon, and M.-Y. Kim	*Does one size fit all A dual double diamond approach to country-specific advantages*	2009	2.85
34	A. Ali, H. Wang, A.N. Khan, A.H. Pitafi, and M.W. Amin	*Exploring the knowledge-focused role of interdependent members on team creative performance*	2019	11.33
34	L. Cui and F. Jiang	*Ownership decisions in Chinese outward FDI: An integrated conceptual framework and research agenda*	2009	2.62
33	M. Pudelko and H. Tenzer	*Subsidiary control in Japanese, German and US multinational corporations: Direct control from headquarters versus indirect control through expatriation*	2013	3.67
33	V. Peltokorpi and L. Clausen	*Linguistic and cultural barriers to intercultural communication in foreign subsidiaries*	2011	3.00
32	B.I.I. Park, A. Giroud, H. Mirza, and J. Whitelock	*Knowledge acquisition and performance: The role of foreign parents in Korean IJVs*	2008	2.29
31	K. Lee and X. He	*The capability of the Samsung group in project execution and vertical integration: Created in Korea, replicated in China*	2009	2.38

*TC* total citations and *TC/Y* Citations per year since initially published, For Calculation of TC/Y, 2021 is considered as present year. In case of publication in 2022, number of periods is considered as 1

## Science mapping of *Asian business and management*

### ABM’s social structure from co-authorship analysis

**Fig. 2:**
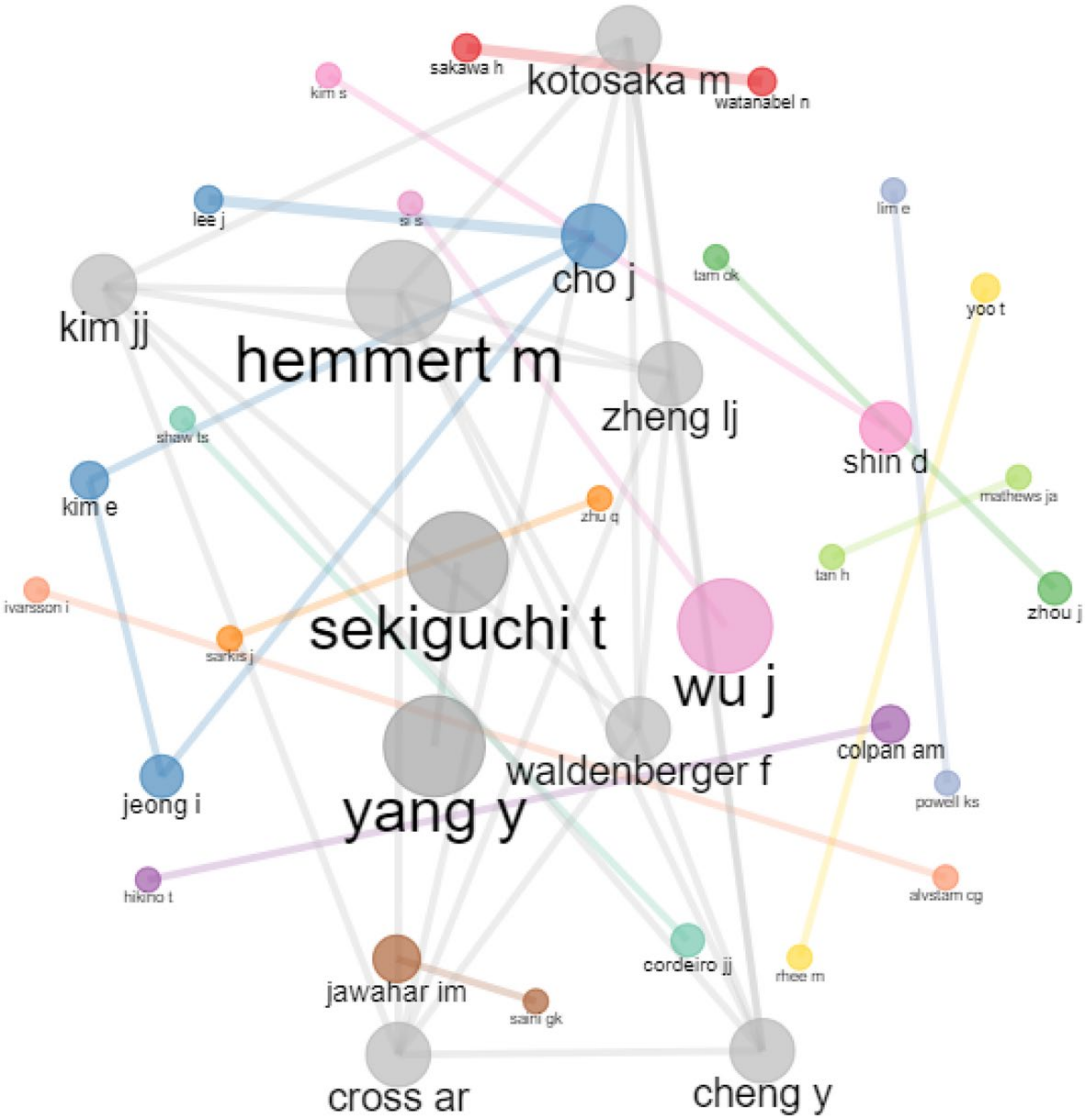
Author co-authorship network. *Note* Threshold for inclusion is minimum number of two edges in the network with 500 nodes

**Fig. 3:**
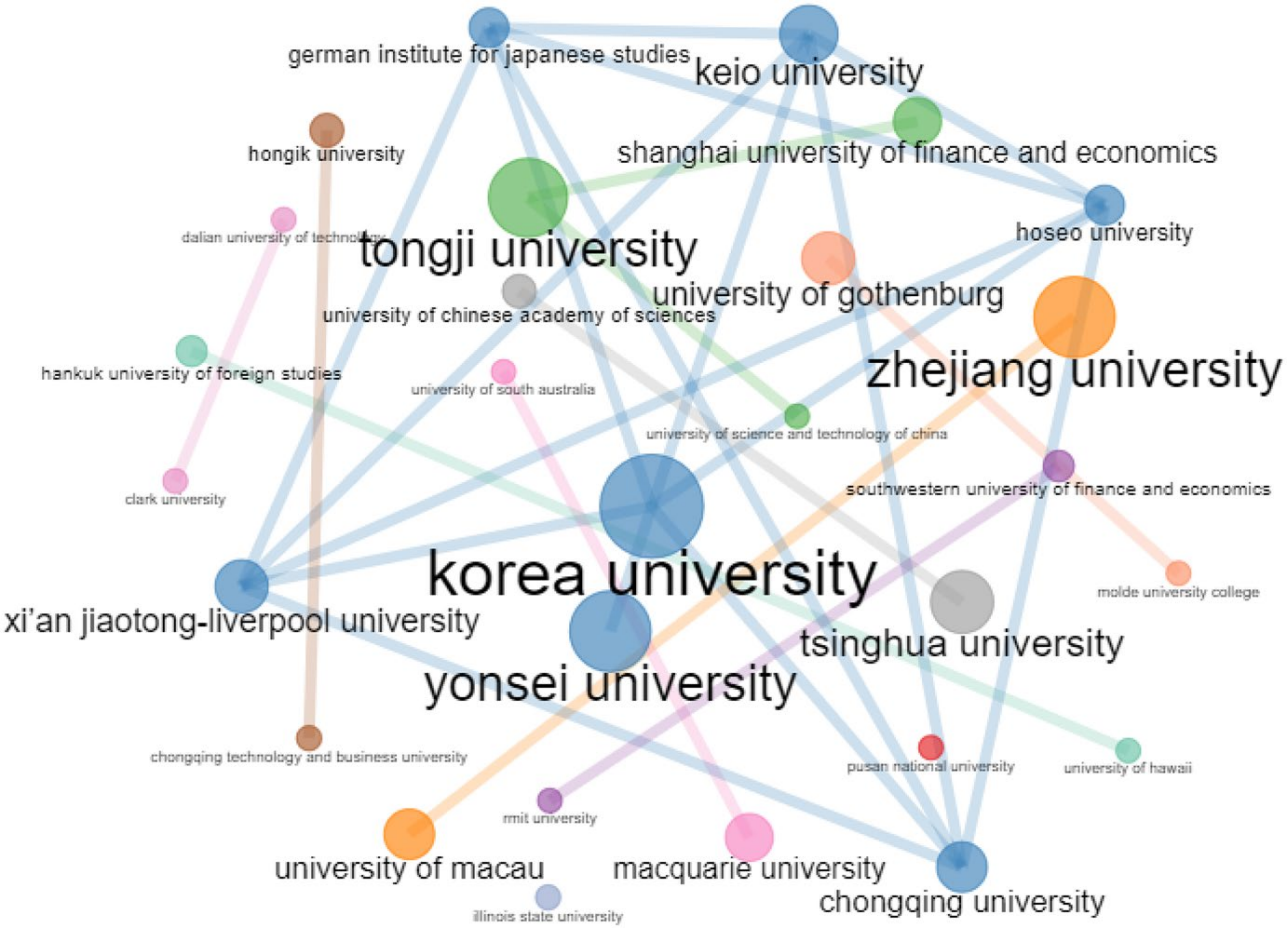
Institution co-authorship network. *Note* Threshold for inclusion is minimum number of two edges in the network with 500 nodes

**Fig. 4:**
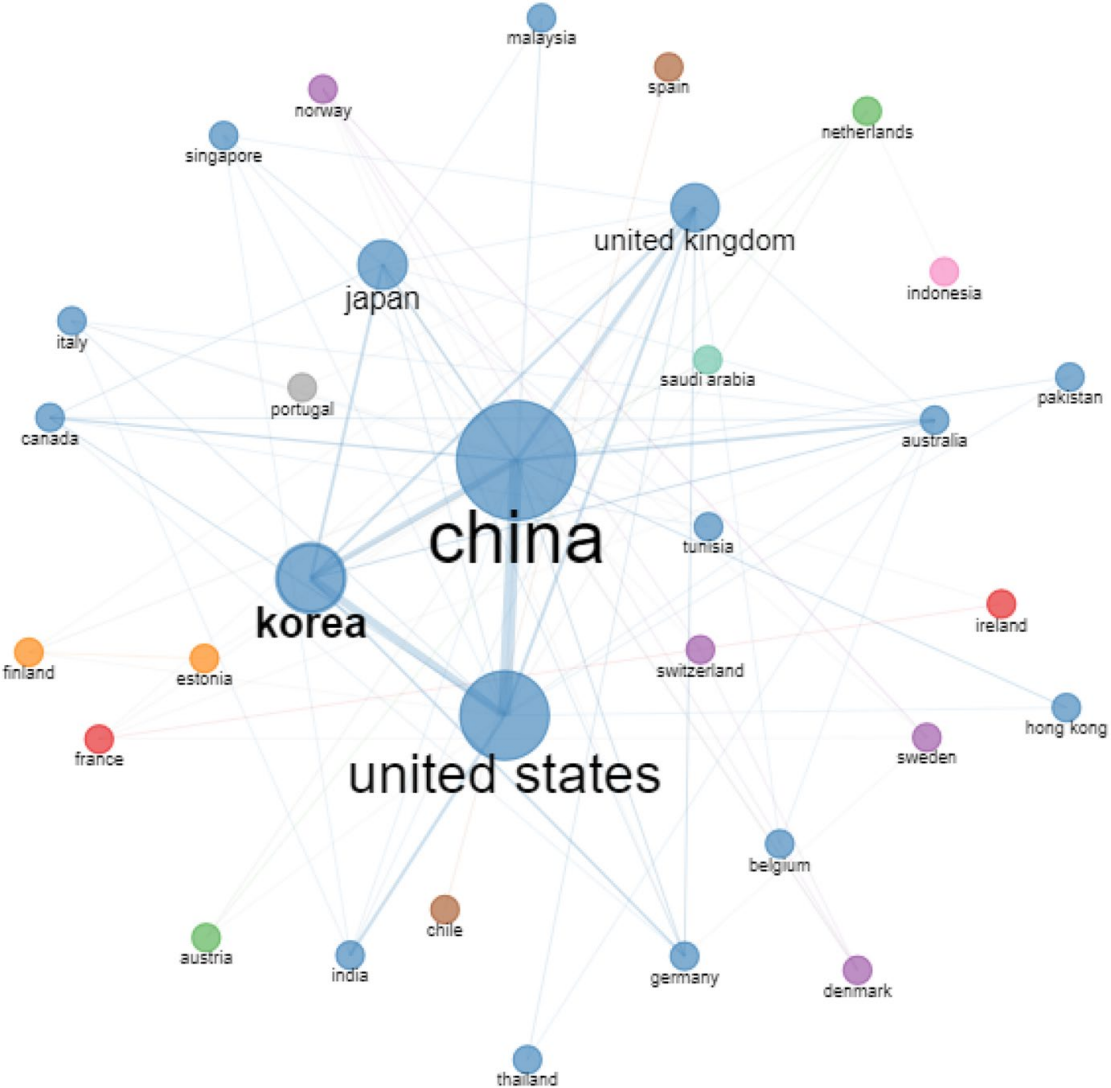
Country co-authorship network. *Note* Threshold for inclusion is minimum number of two edges in the network with 500 nodes

ABM’s social structure is explored by means of a co-authorship analysis of research constituents, and the resultant network analysis and network visualization are provided in Figs. 2, 3 and 4 (RQ4). To simplify interpretation, the nodes in these network maps represent research contributors, while the connections between these nodes represent co-authorship patterns (Dana et al., 2021; Donthu et al., 2022; Kataria et al., 2021). Co-authorship is represented as a cluster of nodes of the same color; the size of the node and thickness of the linkage show the frequency with which they occur (Aria & Cuccurullo, 2017; Donthu et al., 2021).

To begin, we examined the data from single-authored articles in the extracted review corpus of 331 articles to gain a better understanding of the overall status of collaboration across research constituents and found that 20.24% publications (67 articles) were authored by a single author. When this data were broken into two time phrases, single-authored articles accounted for 28.99% (40 out of 138 articles) between 2008–2014 and 13.99% (27 out of 193 articles) between 2015 and 2022. Given the importance and pervasiveness of collaboration in addressing expanding conceptual and methodological complexities in research, it is unsurprising that the number of single-authored articles is steadily falling with each passing year (Donthu et al., 2022). This suggests that multiauthored articles have increased as a percentage of ABM articles, showing that academic collaboration is becoming more prominent in ABM.

Table 7 presents the most important authors, institutions, and countries across different measures of centrality. Four metrics of centrality were used in this review investigation: degree of centrality; betweenness centrality; closeness centrality; and eigen centrality (Badar et al., 2013; Huang et al., 2021). The degree of centrality of a node in a network is defined as the number of relational linkages it possesses (Vázquez-Cano et al., 2022). In contrast, betweenness centrality refers to a node’s ability to connect otherwise isolated groups of nodes (Makkizadeh & Sa’adat, 2017). The third measure, closeness centrality, refers to a node’s proximity to every other node in the network, with nodes having more shortest paths than others in the network, showing their ability to convey data and information across the network with relative ease (Köseoglu et al., 2018). Finally, eigen centrality refers to the importance of a node linking to other highly linked nodes in terms of information flow (Rishiwal & Kumar, 2016).

**Table 7:** Centrality measures of authors, institutions and countries

Rank	Degree of centrality	Betweenness centrality	Closeness centrality	Eigen centrality
*Authors*				
1	T. Sekiguchi	T. Sekiguchi	H. Sakawa	T. Sekiguchi
2	J. Cho	R. Bebenroth	N. Watanabel	J. Wu
3	D. Sutherland	J. Li	Y. Wang	J. Cho
4	F.J. Froese	M. Hemmert	F.L. Cooke	Y. Yang
5	M. Hemmert	A.M. Colpan	A. Giroud	F.J. Froese
6	J. Li	L. Zhang	P. Pananond	K. Lee
7	A.M. Colpan	F.J. Froese	M.H. Ogasavara	X. Wang
8	J. Wu	J. Wu	Y. Hoshino	J.Y. Lee
9	Y. Cheng	D. Sutherland	J. Cho	G. Liu
10	A.R. Cross	J. Cho	I. Jeong	M. Yang
*Institutions*				
1	Korea University	Korea University	Hankuk University of Foreign Studies	Korea University
2	Zhejiang University	Kyoto University	University of Hawaii	Zhejiang University
3	Shanghai University of Finance and Economics	Osaka University	Thammasat University	Keio University
4	Yonsei University	Yonsei University	University of Manchester	Xi’an Jiaotong-Liverpool University
5	Keio University	Hongik University	Osaka University	Kyoto University
6	Tongji University	University of Reading	Keio University	Chongqing University
7	Shanghai Jiao Tong University	Keio University	Korea University	Renmin University of China
8	Chongqing University	Zhejiang University	Kobe University	Shanghai University of Finance and Economics
9	Osaka University	Tongji University	Kyoto University	Yonsei University
10	University of Macau	Shanghai Jiao Tong University	Yonsei University	Seoul National University
*Country*				
1	China	China	Spain	China
2	United States	United Kingdom	Chile	United States
3	United Kingdom	United States	China	United Kingdom
4	South Korea	Japan	United States	South Korea
5	Japan	South Korea	United Kingdom	Japan
6	Australia	Netherlands	Japan	Australia
7	Germany	Australia	Australia	Germany
8	Canada	France	South Korea	Canada
9	Spain	Italy	Germany	Spain
10	Chile	Canada	France	Chile

When considering author aspects (Table 7), Sekiguchi is the most notable author on the metrics of degree of centrality, between centrality, and eigen centrality, while Sakawa is the most notable author on closeness centrality. A similar trend can be found in terms of institutions, with Korea University being the most significant institution on measures of degree of centrality, between centrality, and eigen centrality, and Hankuk University of Foreign Studies being the most notable institution on closeness centrality. Last, in terms of countries, China ranks first on measures of degree of centrality, between centrality, and eigen centrality, while Spain ranks first on metrics of closeness centrality. These findings underscore the engagement of important stakeholders who contribute to ABM.

Following upon the preceding discussion, Fig. 2 demonstrates that there are author groups in ABM that are relatively separated from one another, since scholars prefer to work with the same group frequently. As per Fig. 2, there are nine major clusters of author collaborations with at least two co-authored articles in the network, with noteworthy cross-exchanges occurring between author groups led by Sekiguchi, Yang, and Hemmert. Figure 3 depicts eight key clusters of institutional collaborations when assessed with two co-authored publications in the network and reinforces prior results for Korea University, Yonsei University, Tongji University, and Zhejiang University, among others. The country collaboration network shown in Fig. 4, on the other hand, looks to be less complex than the previous collaboration network, with China in the center and linked to the United States and Korea as major nodes, which appears to be interconnected with other countries. These findings indicate that ABM authors collaborate more actively among institutions than across countries or other teams.

### ABM’s intellectual structure from key co-occurrence analysis and bibliographic coupling

**Fig. 5:**
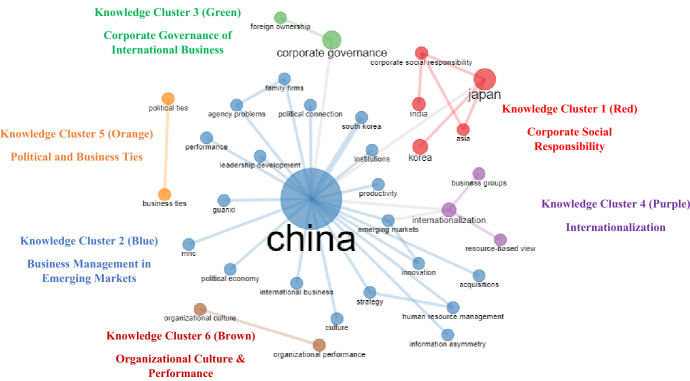
Network representation of major knowledge clusters of ABM

As described in previous sections, an author’s keywords are an adequate depiction of an article’s theme, and their co-occurrence aids to spotting notable trends in any specific field (Gammelgaard et al., 2020; Pattnaik et al., 2021; Varma et al., 2022; Viglia et al., 2022). To determine the intellectual structure of ABM (RQ5), science mapping was done using two standard techniques, i.e., key co-occurrence and bibliographic coupling. The first technique identifies multiple groupings of co-occurring keywords that represent knowledge clusters (or important topics), while the second technique identifies articles inside each discovered knowledge cluster (Aria & Cuccurullo, 2017; Donthu et al., 2021; Sureka et al., 2020). The results of the analyses have been triangulated and are as presented in Fig. 5 and Table 8, demonstrating that papers produced in ABM between 2008 and 2022, since its indexing in Scopus, have centered on six major themes, which are discussed in greater depth below.

**Table 8:** Conceptual Structure of Articles published between 2008 and 2022 (Overall)

Knowledge Cluster (No. of Articles) and Prominent Keyword (Betweenness, Closeness, PageRank)	Top 3 Cited Articles in Each Knowledge Cluster		
	Authors (Year)	Article	TC
**KC1 (42 articles, TC = 346): Corporate Social Responsibility** **Prominent Keywords:** Japan (103.000,0.018,0.049), Corporate Social Responsibility (28.000,0.012,0.039), Korea (0.000,0.012,0.015), India (0.000,0.009,0.015), and Asia (0.000, 0.012,0.026)	M. Pudelko and H. Tenzer (2013)	*Subsidiary control in Japanese, German and US multinational corporations: Direct control from headquarters versus indirect control through expatriation*	33
	B. Amann, J. Jaussaud, and I. Martinez (2012)	*Corporate social responsibility in Japan: Family and non-family business differences and determinants*	29
	C.Y. Chung, S.J. Cho, D. Ryu, and D. Ryu (2019)	*Institutional blockholders and corporate social responsibility*	27
	R.C. Kim and J. Moon (2015)	*Dynamics of corporate social responsibility in Asia: Knowledge and norms*	27
**KC2 (241 Articles, TC = 1865): Business Management in Emerging Markets** **Prominent Keywords:** China (384.500,0.027,0.264), Emerging Markets (1.500,0.016,0.035), South Korea (0.000,0.015,0.024), Innovation (0.000, 0.016, 0.024), Performance (0.000, 0.016,0.024), Family Firms (0.000, 0.016, 0.025), Institutions (0.000,0.015,0.014), Guanxi (0.000,0.015,0.014), Human Resource Management (0.000, 0.016, 0.025), International Business (0.000, 0.015, 0.014), Leadership Development (0.000, 0.015, 0.014), Productivity (0.000, 0.015, 0.014), Strategy (0.000, 0.016, 0.025), Acquisitions (0.000,0.016,0.025), Agency Problems (0.000,0.015,0.014), Culture (0.000, 0.016,0.025), Information Asymmetry (0.000, 0.015,0.014), MNC (0.000,0.015,0.014), Political Connection (0.000, 0.015,0.014), and Political Economy (0.000, 0.015,0.014)	Y. Liu, J.M. Lee, and C. Lee (2020)	*The challenges and opportunities of a global health crisis: the management and business implications of COVID-19 from an Asian perspective*	101
	P. Shapira and J. Wang (2009)	*From lab to market? Strategies and issues in the commercialization of nanotechnology in China*	57
	N.A. Khan, A.N. Khan, and S. Gul (2019)	*Relationship between perception of organizational politics and organizational citizenship behavior: testing a moderated mediation model*	52
	Q. Zhu, J. Sarkis, and Y. Geng (2011)	*Barriers to environmentally friendly clothing production among Chinese apparel companies*	52
**KC3 (16 Articles, TC = 178): Corporate Governance of International Business** **Prominent Keywords:** Corporate Governance (28.000,0.016,0.028) and Foreign Ownership (0.000,0.011,0.016)	C.F. Goh, A. Rasli, and S.-U.-R Khan (2014)	*CEO duality, board independence, corporate governance and firm performance in family firms: Evidence from the manufacturing industry in Malaysia*	21
	D. Ryu, D. Ryu, and J.H. Hwang (2017)	*Corporate governance, product-market competition, and stock returns: Evidence from the Korean market*	20
	H. Aman and P. Nguyen (2012)	*The size and composition of corporate boards in Japan*	19
**KC4 (17 Articles, TC = 158): Internationalization** **Prominent Keywords:** Internationalization (55.000,0.017,0.049), business groups (0.000,0.011,0.015), and resource-based view (0.000,0.011,0.015)	T. Sekiguchi, F. Jintae Froese, and C. Iguchi (2016)	*International human resource management of Japanese multinational corporations: Challenges and future directions*	41
	K. Rong, Y. Lin, B. Li, T. Burström, L. Butel, and J. Yu (2018)	*Business ecosystem research agenda: More dynamic, more embedded, and more internationalized*	28
	D.W. Yiu, F.W., Ng, and X. Ma (2013)	*Business group attributes and internationalization strategy in China*	23
**KC5 (8 Articles, TC = 72): Political and Business Ties** **Prominent Keywords:** Political Ties (0.00,1.00,0.029) and business ties (0.000,1.000,0.029)	Y.-H. Lin, C.-J. Chen, and B.-W. Lin (2014)	*The roles of political and business ties in new ventures: Evidence from China*	28
	F. Robins (2010)	*China: A new kind of mixed economy*	15
	R. Fralich and H. Fan (2018)	*Legislative political connections and CEO compensation in China*	8
**KC6 (7 Articles, TC = 26): Organizational Culture and Performance** **Prominent Keywords:** Organizational Culture (0.00,1.00,0.029) and Organizational Performance (0.000,1.000,0.029)	C.J. Teh, A. Boerhannoeddin, and A. Ismail (2012)	*Organizational culture and performance appraisal process: Effect on organizational citizenship behavior*	15
	K. Friesenbichler and E. Selenko (2017)	*Firm performance in challenging business climates: Does managerial work engagement make a difference?*	5
	L. Zhang, J.A. Parnell, and C. Xiong (2021)	*Market and Nonmarket Strategies (NMS) in China: Performance Payoffs in Turbulent Environments*	4

331 articles were published between 2008 and 2022. Analysis conducted using “co-occurrence network” in Bibliometrix-R

*KC* knowledge cluster, *TC* total citations

Figure 5 is a network diagram depicting the interconnectedness of numerous keywords; thus, several aspects must be considered for the reader’s understanding. In a network visualization, each keyword is represented by a node; the size of the node indicates the frequency with which that keyword appears in the review corpus; a line joining two nodes indicates a co-occurring connection between two keywords; and the thickness of the line between two nodes indicates the degree of co-occurrence (i.e., the thicker the line, the more co-occurrence and vice versa) (Aria & Cuccurullo, 2017; Donthu et al., 2021; Emich et al., 2020). Each node takes on the colour of the cluster to which it belongs, with the largest node in each grouping representing its overarching concept. To further illustrate the importance of keywords in their cluster, we have quantified the nodes and links, as shown in Fig. 5, in terms of betweenness, closeness, and PageRank centrality (as stated in Table 8). The significance of a node in a cluster may be inferred by looking at its betweenness centrality, which is the percentage of the cluster’s shortest paths that go to that node (Barthelemy, 2004; Leydesdorff, 2007). The degree of closeness between two nodes is measured by the inverse of the average distance of the shortest paths to and from that node in the network (Feicheng & Yating, 2014; Sedighi, 2016; Vega-Oliveros et al., 2019). Finally, PageRank centrality analyses the contextual network structure to assign a weight to each node based on the number of incoming connections the node has relative to other nodes that are also considered significant (Donthu et al., 2022; Leydesdorff, 2007; Vega-Oliveros et al., 2019). Table 8 provides the metrics for each keyword in each of the six knowledge clusters, which will be mentioned next to the keyword in parenthesis. The avenues for future research (RQ6) have been underlined in the discussed knowledge cluster for interested researchers to explore further.

#### Cluster 1: corporate social responsibility

With 42 articles, this substantial cluster is the second-largest knowledge cluster and is concentrated on notable keywords, such as Japan, corporate social responsibility, Korea, India, and Asia, based on centrality scores. This cluster, which garnered a total of 346 citations, is also the second-most influential cluster. A majority of articles in this cluster studied and presented effective practices of Asian companies, thus illustrating their self-imposed responsibility toward society in areas such as the environment, economy, employee well-being, and competition ethics. Some articles in the cluster focus on the Japanese economy, remarking on subsidiary control procedures, fostering an entrepreneurship culture, and analysing postacquisition performance, among other topics. This knowledge cluster also offers future research opportunities for the next generation of academics. Companies are beginning to regard their corporate social responsibility as a vehicle to create transformative ideas within their organization and disseminate them throughout the industrial sector as a whole. This trend is an opportunity for academics to examine and describe such corporate dynamics in the Asian context in upcoming issues of ABM. For example, interested researchers might explain the motivation of Asian companies to increase their corporate social responsibility capabilities in order to conceptualise and critique complicated risk-resilience-responsibility arrangements in globally dispersed supply chains (Kim & Moon, 2015).

#### Cluster 2: business management in emerging markets

This knowledge cluster is the largest, with 241 articles cited 1865 times in total, making it the most influential cluster as well. Some of the keywords with high PageRank relevance are China, emerging markets, South Korea, innovation, performance, family firms, human resource management, strategy, acquisitions, and culture, all of which reflect on the central theme of business management strategies in the context of emerging markets. The majority of the articles in this group attempt to illustrate, primarily from a microeconomic vantage point, the procedures involved in managing a company’s integrated commercial operations. These operations include marketing, innovation, production systems, social structures, cross-border acquisitions. Some of these studies in the knowledge cluster have taken into account macroeconomic factors such as cultural identity, political environment, competitive dynamics, and other factors in order to empirically reflect business models, which include their requirement to plan, organise, direct, manage, and control a firm’s resources in order to achieve a company’s objectives. The article “The challenges and opportunities of a global health crisis: The management and business implications of COVID-19 from an Asian perspective” by Liu et al. (2020) is the most-cited (TC = 101) in the cluster, owing to its emphasis on resilience, strategic agility, and entrepreneurship in the frame of reference of the macroeconomic business landscape’s struggle against the COVID-19 pandemic. Despite being the largest cluster, the limitations identified in published articles of this knowledge cluster show the necessity for further investigation providing the technological viewpoint of business management in such an economy. Future studies should also look at ways to safeguard the long-term survival of small- and medium-sized businesses, which make a significant contribution to the economy.

#### Cluster 3: corporate governance of international business

This knowledge cluster includes 16 articles with a maximum of 178 citations and is based on two keywords: corporate governance and foreign ownership. The majority of articles in this knowledge cluster focus on corporate governance activities, which refer to the framework of rules, policies, and processes used to oversee and manage a corporation, especially foreign-owned enterprises. Different boards of directors might have different roles and responsibilities, which have been a focus of several of the articles in this set. These have also shown empirically how poor corporate governance may jeopardise a company’s operations and eventual profitability. One of the most impactful articles in this knowledge cluster is titled “CEO duality, board independence, corporate governance, and firm performance in family firms: Evidence from the manufacturing industry in Malaysia,” as authored by the research team of Goh et al. (2014), which garnered 21 citations. It’s possible that the best corporate governance is unique to each organization and industry, and that imposing universal norms or standards would have little effect on improving policy or productivity. As a consequence, given that the qualitative methodological approach allows the researcher to obtain rich information in this field, more qualitative research is required to assist practitioners on sector-specific effective corporate governance practices.

#### Cluster 4: internationalization

This knowledge cluster comprises 17 articles receiving 158 citations and premised upon three keywords: internationalization; business groups; and resource-based view. Articles in the knowledge cluster that drew mostly on a firm’s resource-based view attempted to provide an explanation for the dynamic interaction among institutions, business groups, and internationalization. Articles in the knowledge cluster aimed to demonstrate how businesses participate in people-related or functional processes that increase firms’ engagement in international marketplaces, such as human resource management, business ecosystems, acquisitions, and subsidiary management. With 41 citations, the article titled “International human resource management of Japanese multinational corporations: Challenges and Future Directions” by Sekiguchi et al. (2016) is the most-cited in the cluster. Researchers with an interest in the topic of internationalization might concentrate their energies on researching the distinct factors that lead to the forward or reverse internationalization of businesses (Feng et al., 2020). While some studies on the outcomes of internationalizing R&D activities have concentrated on the innovative performance of parent and subsidiary firms, a few have also emphasised the enterprise’s financial performance and productivity, which opens up new avenues for researchers.

#### Cluster 5: political and business ties

This knowledge cluster, which consists of two main keywords, i.e., political ties and business ties, includes eight publications that have received 72 citations. These eight articles sought to demonstrate how corporations operating in Asian economies should exercise caution when leveraging commercial and political relationships as well as how they can adapt their political–business ties to changing institutional and market contexts. The article by Lin et al. (2014) titled “The roles of political and business ties in new ventures: Evidence from China” has received the most citations, with 28. Highlighting a possible asymmetric association between business and political ties, interested researchers on the topic can investigate their interaction on product innovation activities, marketing strategies, corporate social responsibilities, and organizational learning behavior, among other organization dynamics.

#### Cluster 6: organizational culture and performance

Knowledge Cluster 6 is the smallest cluster, consisting of just seven articles, which were found via a search for co-occurrence of author keywords in a review corpus of 331 articles. These seven articles garnered 26 citations, with two prominent keywords, i.e., organizational culture and organizational performance. Although certain articles emphasise the possibility of direct links between organizational culture and performance, more research is needed to understand the links between diverse cultural dimensions such as involvement culture, consistency culture, adaptability culture, and mission culture with diverse organizational performance. These relationships may also be investigated in the context of tumultuous periods, such as war scenarios, pandemics, and market dynamics, among other circumstances.

#### Temporal comparison of research clusters

**Fig. 6:**
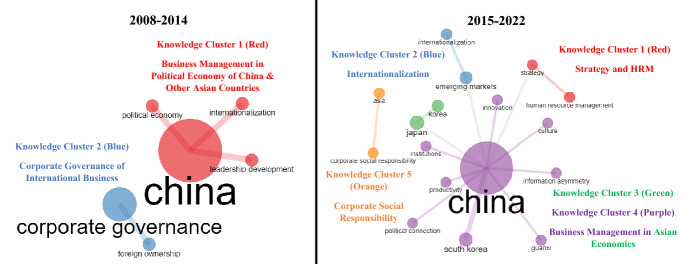
Evolution of major themes in ABM from first time frame (2008–2014) to second time frame (2015–2022)

Despite the fact that six knowledge clusters were identified, showing ABM’s journey from 2008 to 2014 with 331 articles, we ran a temporal analysis over two time periods to identify the most-dominate knowledge clusters in each period (RQ5). Two separate analyses were performed on the 138 articles published in the first time period (2008–2014) and the 193 articles published in the second (2015–2022), the findings of which are presented in Tables 9 and 10, respectively. The data in these tables show that the six aforementioned clusters emerged in some form or another in both separated time phrases. Figure 6 depicts the evolution of ABM’s intellectual structure, revealing the identification of just one distinct cluster in the second time phrase (2015–2022). As seen in Fig. 6 and backed by evidence shown in Table 9, two major knowledge clusters occur in the first time phrase (2008–2014). Among the 138 articles published in ABM between 2008 and 2014, the majority (100 articles) focused on business management in the political economy of China and other Asian countries, while the remaining 38 articles focused on corporate governance for international business. Likewise, referring to Fig. 6 and the information presented in Table 10, it is clear that the five knowledge clusters that have emerged during 2015–2022 are generally closely connected to the six knowledge clusters identified during the aggregate phase (2008–2022). In the aggregate (2008–2022) and sliced time periods (2008–2014, 2015–2022), the thematic cluster predicated on articulating business management in emerging markets emerged as the largest and most influential cluster. The cluster, based on two keywords, i.e., strategy and human resource management, is distinct and gains traction during the second time period.

**Table 9:** Conceptual Structure of Articles published between 2008 and 2014 (First Time Frame)

Knowledge Cluster (No. of Articles) and Prominent Keyword (Betweenness, Closeness, PageRank)	Top 3 Cited Articles in Each Knowledge Cluster		
	Authors (Year)	Article	TC
**KC1 (100 Articles, TC = 1113): Business Management in Political Economy of China and Other Asian Countries** **Prominent Keywords:** China (3.000, 0.333,0.320), Internationalization (0.000,0.200,0.116), Leadership Development (0.000,0.200,0.116), and Political Economy (0.000,0.200,0.116)	P. Shapira and J. Wang (2009)	*From lab to market? Strategies and issues in the commercialization of nanotechnology in China*	57
	Q. Zhu, J. Sarkis, and Y. Geng (2011)	*Barriers to environmentally friendly clothing production among Chinese apparel companies*	52
	J. Mathews (2009)	*China, India and Brazil: Tiger technologies, dragon multinationals and the building of national systems of economic learning*	42
**KC2 (38 Articles, TC = 418): Corporate Governance of International Business** **Prominent Keywords:** Corporate Governance (0.000,1.000,0.167) and Foreign Ownership (0.000,1.000,0.167)	C.F. Goh, A. Rasli, and S.-U.-R Khan (2014)	*CEO duality, board independence, corporate governance and firm performance in family firms: Evidence from the manufacturing industry in Malaysia*	21
	S. Subramanian and V.N. Reddy (2012)	*Corporate governance disclosures and international competitiveness: A study of Indian firms*	11
	T. Yoo and Y. Koh (2014)	*Agent or structure for principal-principal conflicts? Audit firms versus foreign ownership in the Asian context*	9

138 articles were published between 2008 and 2014. Analysis conducted using “co-occurrence network” in Bibliometrix-R

*KC* knowledge cluster, *TC* total citations

**Table 10:** Conceptual Structure of Articles published between 2015 and 2022 (Second Time Frame)

Knowledge Cluster (No. of Articles) and Prominent Keyword (Betweenness, Closeness, PageRank)	Top 3 Cited Articles in Each Knowledge Cluster		
	Authors (Year)	Article	TC
**KC1 (8 Articles, TC = 59): Strategy and HRM** **Prominent Keywords:** Strategy (13.000,0.036,0.061) and Human Resource Management (0.000,0.024,0.035)	F.J. Froese, D. Sutherland, J.Y. Lee, Y. Liu, and Y. Pan (2019)	*Challenges for foreign companies in China: implications for research and practice*	23
	M.P. Maharjan and T. Sekiguchi (2016)	*Human resource management practices at foreign-affiliated companies in least-developed regions: US and Japanese Companies in Nepal*	12
	M. Hemmert (2020)	*Does Korean-style management have a future?*	8
**KC2 (16 Articles, TC = 137): Internationalization** **Prominent Keywords:** Emerging Markets (13.000,0.036,0.061) and Internationalization (0.000,0.024,0.035)	T. Sekiguchi, F. Jintae Froese, and C. Iguchi (2016)	*International human resource management of Japanese multinational corporations: Challenges and future directions*	41
	K. Rong, Y. Lin, B. Li, T. Burström, L. Butel, and J. Yu (2018)	*Business ecosystem research agenda: More dynamic, more embedded, and more internationalized*	28
	J. Cho and J. Lee (2018)	*Internationalization and performance of Korean SMEs: The moderating role of competitive strategy*	16
**KC3&4 (159 Articles, TC = 938): Business Management in Asian Economies** **Prominent Keywords:** Japan (13.000,0.036,0.061), Korea (0.000,0.024,0.035), China (88.000,0.059,0.320); South Korea (0.000,0.033,0.054), Innovation (0.000,0.033,0.032), Guanxi (0.000,0.033,0.032), Institutions (0.000,0.033,0.032), Productivity (0.000,0.033,0.032), Culture (0.000,0.033,0.032), Information Asymmetry (0.000,0.033,0.032), and Political Connection (0.000,0.033,0.032)	Y. Liu, J.M. Lee, and C. Lee (2020)	*The challenges and opportunities of a global health crisis: the management and business implications of COVID-19 from an Asian perspective*	101
	N.A. Khan, A.N. Khan, and S. Gul (2019)	*Relationship between perception of organizational politics and organizational citizenship behavior: testing a moderated mediation model*	52
	J. Wu and S. Si (2018)	*Poverty reduction through entrepreneurship: incentives, social networks, and sustainability*	39
**KC5 (10 Articles, TC = 99): Corporate Social Responsibility** **Prominent Keywords:** Asia (0.000,1.000,0.059) and Corporate Social Responsibility (0.000,1.000,0.059)	C.Y. Chung, S.J. Cho, D. Ryu, and D. Ryu (2019)	*Institutional blockholders and corporate social responsibility*	27
	R.C. Kim and J. Moon (2015)	*Dynamics of corporate social responsibility in Asia: Knowledge and norms*	27
	A. Yu, H.-B. Ding, and H.-M Chung (2015)	*Corporate social responsibility performance in family and non-family firms: The perspective of socio-emotional wealth*	25

193 articles were published between 2015 and 2022. Analysis conducted using “co-occurrence network” in Bibliometrix-R

*KC* knowledge cluster, *TC* total citations

#### Cluster 7: strategy and human resource management

This knowledge cluster of seven articles represents the niche focus of scholarly investigation to emphasise the strategic importance of human resource management within organizations. This cluster of articles has broadened its discussion of individual performance and institutional growth while also providing empirical explanations of the culture and values in human resource management practices. Cluster articles conceptualise the theory of strategic human resource management, where empirical research has presented findings on how organizations may develop and succeed by taking a complete strategy to building the best personnel. Because the topic is still in its early stages in ABM, future researchers may delve deeper into it to reveal additional information about how organizations might be restructured to be adaptable to changing market conditions by assessing the effectiveness of their human resources.

## Discussion

Since its inception in 2002 as a venue for presenting international business research with a focus on the Asian region, ABM has come a long way. To mark this progress, the present study used a number of bibliometric techniques, including co-authorship, keyword co-occurrence, and bibliographic coupling analyses, to investigate trends and essential aspects in the journal’s content. Moreover, this analysis may simultaneously serve as a representation of research on Asian business and management in general.

Our first RQ focused on trends in ABM publishing levels. Compared with the first time period (2010–2014), the number of publications in ABM increased by 39.85% in the second period (2015–2022). The second and third RQs were concerned with identifying ABM research constituents in terms of publications and impact metrics; the results showed that articles, authors, and institutions from China, Japan, and South Korea dominated the performance metrics. Moreover, we observed that the increase of publications was mainly driven by contributions from China. Our fourth RQ examined how often ABM authors collaborated together, revealing that, when teamwork became the norm, the proportion of works by single authors steadily decreased. There was also clear emphasis among ABM contributors on working with authors from China, South Korea, the United States, and the United Kingdom. The fifth RQ sought to ascertain the conceptual structure of ABM articles. Bibliometric analysis revealed six research clusters: corporate social responsibility; business management in emerging markets; corporate governance of international business; internationalization; political–business ties; and organizational culture performance. As a result of the sixth RQ, temporal analysis revealed strategy and human resource management as a seventh research cluster.

## Future research directions

The findings of this bibliometric analysis builds a foundation on which to develop ideas for future research. The results in this study were purely driven by bibliometric analysis. Our editorial view partially confirms the importance of the identified clusters but also offers different, more nuanced perspectives. We also expect that corporate social responsibility, corporate governance, and internationalization have been and will continue to be important topics for future research and practice in Asian business. Corporate social responsibility is an important topic not only for Asian organizations but for all organizations, i.e., a topic of global significance. However, the meaning, practices, and goals of corporate social responsibility may differ substantially between (Western) industrialised and (Asian) developing countries (Sharma, 2019). Thus, more research on corporate social responsibility in Asia is needed.

Despite or even because of COVID-19 and the Russian War in Ukraine, internationalization is expected to remain an important topic for years to come. Future research may investigate whether and how (Asian) multinational enterprises respond to these changed environmental conditions. Moreover, future research should investigate the applicability of existing internationalization theories, develop completely new theories, or creatively combine different theoretical perspectives. For instance, Wu and Vahlne (2022) offer a framework combining the well-established Uppsala internationalization model with the dynamic capabilities perspective to explain a dynamic evolution. Another promising research direction is to combine research on internationalization with other research clusters. For instance, Lee et al. (2022) zoomed in on the role of business groups (corporate governance) and internationalization.

The bibliometric analysis also revealed the increasing relevance of human resource management in Asia. While China and other Asian countries have been used as the world’s factories, many of those countries have moved up the value chain. In line with this upgrading, people and their skills and competencies have become drivers of success. Correspondingly, human resource management and talent management have become key in Asia (Froese et al., 2020; Li et al., 2022). Human resource management in Asia has been influenced by the West and partially developed their own styles (Malik et al., 2022). More empirical research is encouraged to understand the development and effectiveness of human resource management in Asia. While substantial research has examined China and Japan, much less research exists on other countries. We encourage more research on other less-researched Asian countries.

While corporate governance of large Asian organizations has been identified as a major research cluster in the bibliometric analysis of ABM, small- and medium-sized enterprises and entrepreneurship have received much less attention. This is surprising and in contrast with abundant research on entrepreneurship in Western countries. It seems that Asian business research is lagging behind business practice. Hemmert et al. (2022) provided an excellent overview of entrepreneurship research in Asia, concluding that more contextualised research on Asia and on less-studied themes such as culture, entrepreneurial financing, entrepreneurial teams, new venture internationalization, and new venture entrepreneurial intention is warranted. We thus hope this article will inspire more research on Asian business and management.

## Appendix

**Table:**

Variable	Definition
Publications	
Total number of Publications (TP)	The total number of publications (or articles) shows the academic contributions of ABM, its authors, and (or) their affiliations. It is measured as the total number of articles published in any given year of ABM, the total articles contributed by an ABM author, an author’s affiliation or the total publications constituting a bibliographic cluster of ABM Mathematically, TP = $\mathop \sum \limits_{t = 1}^{n} A_{i},$ where *A* = ABM article and *t* = publishing year(s) between 2002 and 2022
Total Cited Publications (TCP)	This variable is an indicator of impactful research covered in the journal. It is defined as the total number of publications (or articles) cited at least once in Scopus Mathematically, TCP = $\mathop \sum \limits_{i = 1}^{n} Number \,of \,article\left( s \right)_{i},$ where *i* = number of citations
Citation Structure	
Total citations (TC)	As a measure of the academic influence, the variable is defined as the sum of the citations accredited to an article, an author, a cluster, etc Mathematically, TC = $\mathop \sum \limits_{i = 0}^{n} A_{i},$ where, *A* = an ABM article, an ABM author, an ABM author affiliation, an ABM country affiliation, or an ABM bibliographic cluster and *i* = citation(s)
Citations per publication (TC/TP)	This variable shows the average number of research to which an ABM article is found influential. It is defined as the average number of citations per publication Mathematically, $\frac{TC}{{TP}},$ where, TC = total citations and TP = total articles
Citations per cited publication (TC/TCP)	As a more accurate measure, the variable is defined as the average number of citations per cited publication Mathematically, TC/TCP = $\frac{TC}{{\mathop \sum \nolimits_{i = 1}^{n} TP_{i} }},$. where, TC = total citations, TP = total articles, *i* = number of citation(s)
*h-index* (*h*)	As a measure of academic influence, the variable is defined as the h number of ABM articles cited at least h times
Number of active years (Y)	As an indicator of research activity, the variable is defined as the number of years in which at least one ABM article was published, or the number of years an ABM author, an author affiliation, or an ABM cluster published at least one article
*Citation per year since publication (C/Y)*	As a more accurate measure, the variable is defined as the average number of citations per total number of active years Mathematically, *C/Y* = $\frac{TC}{Y}$ where, TC = total citations and *Y* = total number of active years since first published
*Total Referred (TR)*	Number of references (TR) denotes the intellectual connection of articles published in ABM to the broad research area in other Journals. Defined as the count of the ABM articles referred by other journals (or source titles)
*Research Geography*	
Single Country	A dummy variable coded 1 for articles following investigation conducted in one country setting and 0 otherwise
Multi Countries	A dummy variable coded 1 for articles following investigation conducted in multi countries setting and 0 otherwise
